# An Innovative Approach to the Dismantlement of a Forensic Psychiatric Hospital in Italy: A Ten-year Impact Evaluation

**DOI:** 10.2174/18740179-v18-e221221-2022-11

**Published:** 2023-01-01

**Authors:** Liliana Leone, Gaetano Giunta, Gaspare Motta, Giancarlo Cavallaro, Lucia Martinez, Angelo Righetti

**Affiliations:** 1 CEVAS Centre of Research and Evaluation, Roma, Italy; 2 Community Foundation of Messina o.n.l.u.s., Messina, Italy; 3 Department of Secretary General, Fondazione di Comunità di Messina O.n.l.u.s., Italy; 4 Mental Health Department, ASP 5, Messina, Italy; 5 Consortium SO.LE. Solidarity and Energy, Messina, Italy; 6 Istat Italian National Institute of Statistics, Roma, Italy; 7 Scientific Committee- Community Foundation of Messina o.n.l.u.s., Messina, Italy

**Keywords:** Job placement, International classification of functioning, Forensic hospital, Capability approach, Cost analysis, Deinstitutionalization

## Abstract

**Aims:**

This study aimed to evaluate the impacts of a pilot project concerning the closure of a Forensic Psychiatric Hospital (FPH) inspired by Human Development Theory and the Capability Approach.

**Background:**

The dismantlement of the FPH of Barcellona Pozzo di Gotto (Sicily Region in Italy) began in 2010 with the pilot project Luce é Libertà and ended in 2017. With the closure of six FPHs, Italy officially became the first country worldwide to close such institutions. After the closure of FPHs, some critical issues emerged, and the debate shifted to developing small-scale facilities and residences for the execution of security measures (RESM). However, few studies have provided results on the cohort of patients discharged from FPHs.

**Objective:**

Following are the objectives of this study: a) Assessing the effectiveness of the pilot project in terms of better functioning accordingly to the Classification of Functioning of Disability and Health (ICF) framework, social and labour insertion, health conditions, level of dangerousness to other, rate of readmission in forensic services, b) cost analysis, and c) describing how the CA has been applied and translated into methodological and administrative devices and concrete intervention strategies.

**Methods:**

A pre-post evaluation design was performed with a comparison between the intervention and the control group for the healthcare cost analysis. Data were collected from 2010 to 2019 at three points: T0) as a baseline, T1 and T2) for the follow-up. The instruments are a structured questionnaire, the Scale HoNOS Secure, 4 sub-scales of ICF (Activity and participation dimensions: sociality, culture, and knowledge, daily life, income, and work) (Cronbach’s Alpha from 0.76 to 0.94), and n.20 interviews with key stakeholders and beneficiaries.

**Results:**

Main results include a) the discharge of 55 patients through the use of a person-centered approach and the Personal Capability Budget (PCB), b) the expansion of substantial freedom of choice and the improvement of ICF score (t-test Sig. <, 02), c) the reduction of the risk for others and themselves (Mean Diff. -2,15 Sig. .000), d) at T2 42% of beneficiaries achieved a job placement and 36% were living in one's own home, e) at T2 the need of security measures has reduced from the initial 70% to 6.8%, and f) reduction of the healthcare costs from the fourth year onwards.

**Conclusion:**

Indications emerge to support processes of deinstitutionalisation and capabilities expansion through innovative models, a person-centered approach supported by PCBs, social finance, and social impact investments.

## 1 INTRODUCTION

In 2011, Italy initiated the definitive closure of all six maximum-security Forensic Psychiatric Hospitals (FPHs) that provided treatment and custody to mentally ill offenders [1-4] deemed not guilty by reason of insanity and socially dangerous and to patients who became mentally ill during incarceration. These custodial institutions confined mentally ill persons on indeterminate sentences, and the quality of mental healthcare was seriously unsatisfactory, thus depriving them of all civil rights [5]. The definitive closure of the FPHs is the result of ideas inspired by the struggles of “anti-psychiatry”, and “democratic psychiatry” [6] and represents the final step of the reform (Law 180/1978 or “Basaglia Law”).

It was confirmed by the Decree-law number 1/2014, and with the discharge of the last patients at the FPH of Barcellona Pozzo di Gotto, in February 2015, Italy officially became the first country worldwide to close forensic psychiatric hospitals. A few years after the closure of the Italian FPHs, some critical issues emerged concerning the development at a regional level of small-scale mental health facilities and services and possible treatment pathways in and out of institutionalising settings, such as the residences for the execution of security measures (Residenze per la Esecuzione della Misura di Sicurezza, REMS) [7]. However, few studies provided the results of a cohort of patients discharged from forensic hospitals using a prospective longitudinal design [8-11]. According to Barbui and Saraceno [5], there is still a shortage of evidence about the effectiveness of various legal frameworks among the EU Member States. Finally, there is a lack of data in the FPH setting, and no study used ICF to assess the effectiveness of the intervention with ex-patients inside an FPH [12].

The pilot project Luce è Libertà (Light is Freedom from 2010 to 2014) was aimed to discuss the dismantlement of FPH of Barcelona Pozzo di Gotto (Messina, Sicily Region), where serious human rights violations were reported by a parliamentary commission. The project was built in partnership with the Mental Health Department of the Provincial Health Authority (ASP ME) of Messina, the ULEPE (Local Office for External Criminal Execution), the centre for social innovation Ecos-Med Social Cooperative Society., and a network of social consortia in Sicily. It was co-financed by Cassa delle Ammende of the Ministry of Justice for a value of € 3,894,886 and by the local partners for a start-up amount of € 1,248,520. The project aimed to experiment with a community welfare model structurally intertwined with forms of the productive green economy. The theoretical model underlying the pilot project was based on the human development paradigm and, following the Capability Approach (CA), addresses the expansion of beneficiaries’ capabilities [13-15]. The main goal was to simultaneously promote socio-economic systems generating alternatives on the main areas of the beneficiaries' human functioning and expanding their substantial freedoms. The Luce è Libertà project used a person-centered approach, guaranteeing each beneficiary a Personal Capability Budget (PCB), which represents the stock of resources on which to base the re-appropriation of their civil rights at an individual, social and community level [16]. The mutualization of PCBs has made it possible to set up a dedicated fund at the Community Foundation of Messina (CFM) aimed at self-financing Luce and Libertà over the long term (20 years after the start-up).

The impact assessment of the pilot project Luce è Libertà was commissioned to CEVAS by the CFM [17]. The main hypothesis was that the activation of inclusive welfare models based on the protection of rights and the promotion of solidarity bonds in micro-contexts characterized by a high level of social capital at the inter-organizational level offers more opportunities and could trigger mechanisms of freedom expansion and thus guarantee positive outcomes. Expected outcomes were the effective process of social and labour insertion, better health conditions, a low level of danger to others, a lower rate of readmission in forensic services after discharge, lower costs for the national health system, and increased people's substantial freedoms.

## 2 MATERIALS AND METHODS

To compare the process of beneficiaries' capability expansion over time, the pre-post comparison of preferred abilities (*e.g*., income and employment, housing, sociability and affectivity, health and knowledge) was made as the focus of interest [14].

The research aimed to answer the following evaluation questions:

1. After the exit from the FPH, is there an improvement in the welfare conditions of the beneficiaries of the project? Are risk factors and the need for control measures reduced?

2. Does the 'parachute' mechanism work, *i.e*., the protection system given by the activation of formal and informal local support networks, in case the subjects experience phases of crisis? Is recidivism (re-entry to the penal area) reduced?

3. Is the model more or less efficient than the standard model based on hospitalisation in protected healthcare residential structures or therapeutic communities?

The pre-post study design collected data at three points: T0) baseline inside the FPH along the period May 2010 to November 2012, T1) at 20 months follow-up in the period 2012-2013, and T2) a follow-up in December 2019. Ethical approval for the study was granted by a local ethics committee. All participants signed an informed consent form and voluntarily accepted to be involved in the project and agreed to use the PCB as a common, sharing funding for the purpose of productive investment. Due to the yield generated, the social benefits (e.g., labour insertion, social support network, and social housing) have been extended for over 20 years. During the administration of the HoNOS Secure [18] and ICF tests, data from 52 subjects were recorded. Those of a new subsequent insertion without detection at T0 were missing.

The efficiency of the model was calculated by comparing the direct healthcare costs incurred for the beneficiaries of Luce è Libertà with the standard healthcare costs. At the time of the survey, the per capita and per diem fees of social health or residential healthcare facilities for psychiatric patients varied between 130 to 200 euros.

The people who died shortly after the start of the project were excluded from the analysis.

### 2.1 Instruments to Collect Data

Data collection was mainly carried out through a structured questionnaire completed in the pre- and post-version by trained health workers of the Mental Health Department ASP-ME5. It included 124 structured questions as follows: a section with personal information (13 questions: age, marital status, employment, education, *etc*.), psychiatric history, legal data (year of the crime, type of restriction, date, and duration of stay in the FPH, definitive/provisional safety measures and timing), psycho-pathological data (17 questions: diagnosis, history, drugs and therapy, other concomitant pathologies), and social conditions (11 questions: case manager references, job placements, references to the FPH's background, type and frequency of contacts). The Health of the Nation Outcome Scale for Users of Secure and Forensic Services (HoNOS) was administered together with a shortened version of the ICF consisting of 19 items.

The HoNOS-secure (Vers 2b St Andrew) is a structured judgment tool that consists of two scales. The first scale includes 7 items that refer to the risk for oneself, others, and from the others to the level of protection and accompaniment necessary for compliance with the rules and clinical risk. The summary index of HoNOS-secure related to the aspect of mental and physical health conditions and problems in daily life activities includes only 8 of the original 12 items (Items 1, 2, 4, 5, 6, 8, 9) excluding those with over 10% of non-responses at T0 during the detention in the FPH. Scoring ranges from 0 (no risk) to 4.

The second part of the questionnaire includes some items from the International Classification of Functioning, Disability, and Health (ICF) developed in 2001 by the World Health Organization. The ICF items correspond to four dimensions of the Activity Limitation and Participation Restriction, reflecting the areas of empowerment (capability dimensions) also used in the definition of the personalised intervention plans developed through the PCB. The ICF uses two constructs: (a) a person's ability to perform an activity in a standard environment called ‘Capacity qualifier’ and (b) a person's actual performance in performing an activity in his natural environment. Therefore, the performance qualifier (P) represents a measure of an individual's 'functioning', similar to the meaning given by Amartya Sen's Capability Approach. The qualifications/scores represent the limitation or restriction levels and allow us to denote the seriousness of the problem (range 0-4)

A qualitative investigation was carried out through the realization of No. 20 unstructured interviews (No. 8 to patients and No. 12 to project operators and managers). The objectives of the interviews were: 1) understanding the concrete ways of using the personal health budget, the PCB, starting from the management of the numerous 'critical events' (e.g., how to avoid and contain re-entry, buffer function with magistracy, *etc*.), 2) identifying strategies adopted to increase the 'freedoms and offer more opportunities' (primarily housing and job), and 3) analysing the sustainability of the solutions adopted by the project.

All the interviews were recorded, transcribed, and the contents were codified. An in-depth content analysis of the interviews was published in the first evaluation report [19].

### 2.2 Statistical Analysis

The statistical analysis followed two steps:

(a) Construction of some synthetic indexes for HoNOS-secure and the ICF sub-scales. The internal coherence of the scales was measured using Cronbach’s Alpha coefficient, and any reductions in the variables to be entered were decided.

(b) Comparison between the average t-test of the pairs of items between the first and the second survey to highlight the areas in which the significant changes occurred.

There are 39 ICF items, with both the scores for performance and capacity, that are discussed in this analysis (Table **1**). The limitation or restriction levels range from 0, no problem, to 4, complete or deep problem. They have been summarized, with an additive procedure, by defining four sub-scales (Cronbach Alpha coef. from .76 to .94) related to the dimensions used in the personalised projects: Sociality Scale (composed of 8 items), Culture and Knowledge Application Scale (n.10 Items), Living and Daily Life Scale (n.16 items), and Income and Work Scale (n. five items). Therefore, each of the 4 scales further provides four synthetic indicators: a pre- and post-performance measurement (T0 and T1) and a pre- and post-capacity measurement. The scales are then standardized, and the original values reflect the statistics used to build the scales.

## 3 RESULTS

The sample of beneficiaries constituted 55 male subjects with an average age of 45 years: 32% of young people between 24 and 39 years of age, 39% with an average age between 40-50 years, and the remaining 29% of mature adults between 51 and 64 years old. Participation in the project was proposed to all patients of the FPH who had the extension of the measure and, therefore, had experienced greater failures in previous attempts to discharge. The length of stay within the FPH varied, in the first survey (T0), between 2 and 25 years, with only 12 people that remained less than two years. The main crimes committed were: personal injury (12), murder (10), attempted crime (8), theft or minor offenses, such as attempted robbery, and non-compliance with restrictive provisions. The legal position of 48% of the patients at T0 was ‘security measures’ or ‘sentence discounts’, while other beneficiaries were definitively discharged (with final license).

The two HoNOS-secure scales (Table **2**) indicate significant improvements from pre- to post-discharge (T0-T1), ranging from -2.15 points for the risk assessment scale, involving (b) reduction in social risk for others and themselves to -1.12 points for Health Scale (a) due to the reduction of mental problems and hyperactive behaviours (Student t-test paired differences; Sig, 000).

The most significant improvements concern the lower need for protection of residences (20 people out of 52), the lower personal need for protection and assistance (20 cases), and accompaniment during licensing or exiting (29 cases). A quarter of the sample also recorded an improvement concerning clinical risk and its management.

The behaviours showing the greatest changes (Table **3**) are related to the basic and complex activities of daily life (self-care, washing, dressing, using money, organizing free time, shopping), those related to living conditions such as housing and the availability of money to satisfy basic needs (Items 10, 11, 12).

From 2011 to January 2020, 10 deaths were reported (18.2% of the sample). Within the first two years, 2 people died while they were in a high-intensity therapeutic community; 3 people died at their homes 6-8 years after their discharge due to serious health problems. Following were the major contributing causes of death, continuous and uninterrupted intake, often with high doses, of antipsychotic drugs, the presence of non-communicable diseases, and addictions.

### 3.1 Social Functioning

To analyse the social functioning of the subjects enrolled in the project, the ICF provides a method to record the level of impairment or limitation of the person in the domain of ‘Activity and Participation, using performance and capacity qualifiers. Table **4** illustrates the results of the comparison between pre- and post-survey (T0 and T1). The average value of each scale is highlighted relative to the performances (called P0-P1) and the capacities (C0-C1), and the decrease in the indicator value denotes a reduction in the problematic condition. For all the ICF sub-scales, a significant improvement was noted in the second survey after leaving the FPH (T1) with a more intense variation regarding performance, indicating a reduction in problematic conditions. The variation was found to be statistically significant for all the aspects (Sig., 021/ Sig., 000 t-tests), except for capacity related to the area of income and employment (-, 37 Liv Sig., 06), which improved modestly.

The items of the ICF Sociality Area Scale highlighting the improvements are related to informal and formal relationships (49% of the subjects) (communication, community life, recreational aspects, and political life).

The relational dimension and the central role of affectivity also emerged repeatedly in the interviews. Moreover, meaningful relationships were also developed between co-workers and sometimes between former FPH patients. The community of Barcellona Pozzo di Gotto has been able to welcome several former FPH patients.

As stated by an E.R. and a former FPH: “*Now, I'm completely free, and then I say 'I'm leaving for xxx (home town), then I think about the people I would leave here, the relationships I have with the people in Barcelona*.”

It was found that practitioners often developed warm and lasting personal relationships with former FPH patients providing a social support network.

As stated by a case manager and a former FPH professional educator:

“*Every week, I have an appointment to have coffee with xxx, a former patient of the forensic hospital I met more than a decade ago when I worked there. It is a regular appointment that I never forget.” Now xxx is retired, he lives alone in Messina (…), and the territorial services still refer him to me*”.

However, the closest relatives became minor facilitators even in discharge from the FPH. Considering the performance regarding the “Housing and Daily Life Scale,” an improvement was observed (Mean -4,42) in 24 cases out of 53.

Moreover, there was an improvement in the Scale Culture and Knowledge (14 people) due to the attendance at short training courses or workshops (e.g., photovoltaic system maintenance group). The cognitive skill with the greatest benefits was found to be the problem-solving skill (26 people).

In particular, on the item of paid employment, more than half of the sample (34 people) recorded improvements. Important progress was also reported in economic self-sufficiency (22 people).

A strong correspondence emerged between wishes, skills, expectations, and the type of job placement realised. Patients declared themselves satisfied with the employment.

As stated by the MM, a former FPH patient:

“*I have always worked; this is a new job *[*he is working as a cook*].* I feel good, I work, I get paid, better than this is impossible, especially today when there is no work*”.

The existence of cooperatives in the social district, operating in very different areas but following the logic of the system, has greatly expanded the possible alternatives for people (which means expanding freedom of choice) and the possibilities for fruitful encounters between personal expectations and desires and real opportunities. Moreover, according to the CFM, project manager of GG, as this is a long-term project, individual processes can be accompanied and facilitated while respecting the timeframe of each person and the different cooperative teams”.

Concerning housing, between T0 and T1, the following changes have been observed: 3 people were living alone, 2 were living in a group apartment without the permanent presence of socio-health workers, 10 people were in a low protection structure (housing community, family houses), and 12 were in high protection structures.

In January 2013 (T1), the security measure was revoked for 9 beneficiaries; most beneficiaries benefited from alternative measures to internment, and only 12.5%, had returned to the FPH following the violation of the provisions and revocation of the final license.

Between T0 and T2, 42% of beneficiaries achieved a job placement, and at T2, about one out of three was employed in social cooperatives within or outside the Sicily region. In all cases with job placements, there was income support from the Community Foundation of Messina, and in 6 more cases, this support was for the rental costs of the house.

In the last follow-up (T2) of December 2019 (Table **5**), almost ten years after the start of the project, the need for residential structures with high control intensity (REMS) or prison (1 case) reduced from the initial 70%, to 6.8%. Moreover, 36,4% of the beneficiaries, excluding the people who died, lived in their homes.

#### 3.1.1 Efficiency Analysis and Costs Estimation for Residential Healthcare

The beneficiaries of Luce è Libertà in the fourth year from the operational start-up of the pilot project (46 months from December 2011 to September 2015) remained outside the FPH for for an average time of 36 months, that is 79% of the time. In the first 2-3 years, there were continuous changes in housing conditions and restrictive measures, and 16 people were completely in charge of the project Luce è Libertà (from 1 to 46 months; Mean value 18); 20% of the time they were at home, 44% of the time was spent in a therapeutic community, and the rest in low-intensity healthcare structures. At the end of 2015, there were 21 people that still needed intensive healthcare in a therapeutic community (TC). Considering the fees of three different residential structures at low, moderate, and high intensity of healthcare and protection, after 4 years, the total expenditure for residential healthcare services addressed to the project beneficiaries was about € 8.585.000. However, in the analysis, the positive socio-economic externalities determined by the project were not taken into account. This figure corresponds to 21% of saving from the theoretical expenditure that would have been incurred if the same patients were followed only by TCs, excluding 6% of people that would have returned home and the amount of initial budget cofounded by Cassa delle Ammende. We considered that in Sicily, the length of stay in residential facilities in 2018 was twice the national average [20].

## 4 DISCUSSION

Results demonstrated that improvements in mental health were statistically significant in the follow-up period (T1) after the discharge from the FPH, even for patients with a long institutionalisation and serious mental illness.

The need for high control structures (FPH or REMS) along the first two years from the discharge decreased from an initial baseline of 70% (T0) to 12% (T1), reaching 6.7% after the second follow-up (T2). This shows that the 'parachute' mechanism, the protection system activated by the project, was effective. Dangerousness and risk to others is the key criterion for forensic services for evidence-based psychiatry [21]. Furthermore, the study used a structured professional judgment tool (HoNOS-secure) and demonstrated a statistically significant improvement in the criterion (t-test P < 0,000; C.I. 95%).

At the first follow-up (T1), 11.4% of beneficiaries lived alone or with their family, while at T2, the percentage increased, reaching 42%. Employment is a key factor in enhancing the mental health and social inclusion of people with severe mental illness. It is associated with fewer re-hospitalisation episodes [22] and can be a decisive element for the development of a feeling of self-esteem and the attainment of a minimum amount of social status [23]. Therefore, the high percentage of integration in the workplace (42% of the sample in 8 years) should be considered a very positive outcome.

A critical issue, unfortunately, was the high mortality (18.2% of the sample) from the initial discharges from FPH and the early death (average age at death 49). Most people died two years after the discharge. Literature confirmed higher Standardized Mortality Ratios (SMR 2,47 for males) in psychiatric hospitals with a higher rate after discharge [24].

In line with the Capability Approach (CA) [13, 14], the aspect on which we sought to make judgments about the effectiveness of the personalized intervention was the increase in people's substantive freedoms, which is a key aspect of CA that is underrepresented in assessments of an individual's well-being [25]. Taking into consideration the point of view and evaluations of the users and health workers involved in mental health services, the management of the personalised intervention plan is consistent with both the UN Convention on the Rights of Persons with Disabilities (CRPD) [26] and the Capability Approach.

One of the project peculiarities is the use of the device PCB accompanied by the discharge from the FPH and the long-term support of all the beneficiaries. From a technical point of view, PCBs are very innovative. The PCBs have not been transformed into a flow of services (e.g., fees for therapeutic communities, resources directly expendable by people) as commonly happens, even within the personal health budget for mental health [27-29], but represent a 'stock of resources' mutualized and capitalized for productive investments and aimed at supporting the long-term inclusive social economies managed within the district, promoting further entrepreneurial initiatives for providing job or guaranteeing forms of social housing.

Moreover, the fourth year represented the breakeven point of the project, and from then onwards, the capital invested in the project began to generate economic returns that continued to support interventions on various dimensions, such as housing, work, and social issues.

It has been observed that the reduction in health care costs was mainly due to the moderate use of residential facilities and REMS and the strengthening of care and social inclusion processes at the community level. One of the indirect positive impacts of the project was the development of guidelines and programs by the Sicily region aimed at supporting community-based health and social inclusion of all patients discharged from FPH through personalised treatment projects supported by health budgets.

The creation of PCBs could be guaranteed by financial programs of social impact and, therefore, not weighting on public spending, thus making the transition from inefficient welfare models to much more effective and efficient community welfare models possible. For instance, through the partnership of social finance partners, co-managing entities could establish and guarantee the management of customized projects for at least ten years.

The lack of measurement of change through scientific methods, according to Carta and colleagues [30], in Italy represents a critical point that may compromise the development of knowledge coming from one of the world’s most important experiences of humanitarian approach to mental health [31]. For this reason, a reduced version of four ICF sub-scales was built to assess the social and health performances and functions related to the discharge from the FPH within an impact evaluation design, along with diagnostic purposes and clinical practice [32]. The full version of ICF covers 300 pages dealing with 1,413 items, and according to Helander [33], the chief medical officer of the Rehabilitation Program of the WHO, it is too complex and nearly impossible to use for program evaluation purposes. However, as suggested by Bickenbach [34], the Capability Approach can be combined with the ICF tool.

Recently, a research project conducted in the region of Sardinia (Italy) showed that mental health workers, compared to other workers in health sectors, are the most satisfied and optimistic about the quality of mental health care provided and have respect for users' rights, confirming the validity of the Italian community model for mental health [35-37]. However, the score for satisfaction with resources was found to be low, confirming a progressive depletion of resources for mental health care in Italy [37] and the need for accurate monitoring of the costs of different health services and intervention models.

A recent monitoring report on the Italian FPHs and the role of REMS [38] concluded that the relationships between the judiciary and the mental health departments of the local health authorities in the period from 2015 to 2019 became worse. Moreover, the future of the REMS models should be investigated, and other housing solutions could even replace them. Some measures for the conversion of structures and organizational models, with a clear distinction between security policies and health care or psychiatric rehabilitation functions [39], are still needed. The results of the pilot project Luce è Libertà could partially suggest some viable solutions to meet these needs.

The main limitation of our study is the lack of a complete monitoring system, and therefore of microdata at the national level, about the healthcare paths after the discharge of the ex-patients from the six FPHs. This shortcoming flawed the comparison among different models of intervention and their standard costs. A further weakness of the evaluation design is linked to the lack of direct interviews with the beneficiaries five years after the discharge. Many difficulties would have to be overcome to get in contact with and re-interview people in charge of several mental health departments and within therapeutic communities spread over many provinces.

## CONCLUSION

The Italian reform of psychiatric hospitals, with the “pioneering” measures that led to the closure of FPHs, has been considered a revolutionary trait [1] and a choice of 'civilization,' placing this strategy under the scrutiny of health professionals and policymakers in other countries of the world, which are still founded, to varying degrees, on a more restrictive institutionalising approach.

This study provided an impact evaluation of an innovative model developed by the project Luce è Libertà, with a sample of psychiatric patients discharged from one out of six Italian FPHs with a very long history of mental illness and 'detention'. The study addressed the need to reduce the paucity of empirical research around the closure of FPHs within the reform of the Italian mental health care system [29, 39] and, partially, to test the effectiveness of the Italian model of care [1].

An indirect positive impact of the project was the generation of social capital and financial resources used to support the development of inclusive socio-economic systems [40], which continue, after 10 years, to provide an enabling environment for social and work placements of former FPH patients and people with severe psychiatric disorders.

## Figures and Tables

**Table 1:** Internal consistency of four ICF scales in the first survey (T0) and items included for performance (P) and capacity (C) qualifiers.

**Scales**	**Cronbach’s Alpha**		**Num. of Items**	**ICF Activity and Participation Dimension/items Included**
	P0	C0	-	-
Sociality	0,76	0,84	8	d740_Formal relationships; d750_Informal social relationships; d310_ Communicating with -- receiving -- spoken messages; d330_Speaking; d350_Conversation; d910_Community life; d920_Recreation and leisure; d950_ Political life and citizenship
Culture and knowledge	0,91	0,93	10	d810_Informal education; **d825**_Vocational training; **d160**_Focusing attention; d163_Thinking; **d166**_Reading; **d170**Writing; **d172**_Calcolating; **d177**_Making decisions; d175_Solving problems; **d199**_ Learning and applying knowledge, unspecified
Daily life	0,94	0,92	16	**d650**_ Caring for household objects; d510_Washing oneself; d520_ Caring for body parts (brushing teeth, shaving, grooming, etc.); d570_Looking after one’s health; d550_Eating; d630_ Preparation of meals (cooking, etc.); d530_ Toileting; **d210_**Undertaking a single task; **d230**.1_Managing daily routine; d640_Doing housework; d620_ Acquisition of goods and services (shopping, *etc*.); d660_Assisting others; **d155**_ Acquiring skills; **d240.1**_Handling stress; d450_Walking; d440_ Fine hand use (picking up, grasping)
Income and work	0,88	0,9	5	**d610**_ Acquiring a place to live; d870 Economic self-sufficiency; **d845**_ Acquiring, keeping and terminating a job; d850_Remunerative employment; **d865**_ Complex economic transactions

**Table 2:** Differences at T0 and T1 on two HoNOS-Secure scales of Student's t-test index.

-	-	-	**Paired Differences**					-	-	-
-	**Mean T0**	**Mean T1**	-	-	-	**95% C.I. of Diff.**		**t**	**df**	**Sig (*2-tailed)***
**-**	**Pre**	**Post**	**Mean Diff**	**Std. Dev**	**Std. Err Mean**	**Lower**	**Upper**	**-**	**-**	**-**
(a) HoNOS Health Conditions Scale T0 - T1	9,00	7,88	-1,12	1,68	0,24	0,64	1,59	4,75	50	.000
(b) HoNOS Risk Assessment Scale T0- T1	10,27	8,12	-2,15	1,97	0,27	1,60	2,70	7,87	51	.000

**Table 3:** HoNOS-Secure health scale: Items and mean values at T0 and T1.

**Items**	**T0** **Mean Value**	**T1** **Mean Value**
1. Overactive, aggressive, disruptive, or agitated behaviour	1.00	0.90
2. Non-accidental self-injury	0.40	0.30
3. Problem drinking or drug taking	0.80	0.80
4. Cognitive problems	1.40	1.20
5. Physical illness or disability problems	0.80	0.80
6. Problems with hallucinations and delusions	1.20	1.00
7. Problems with depressed mood	1.10	0.90
8. Other mental and behavioural problems	1.10	1.10
9. Problems with relationships	2.00	1.70
10. Problems with activities of daily living	2.20	1.90
11. Problems with living conditions	2.20	1.80
12. Problems with occupations and activities	2.20	1.80

**Table 4:** Paired samples t-test pre-post differences of 4 ICF scales performance and capacity.

-	-	-			**95% Conf.Inter.Diff**		**Paired Diff.**		
	**Scales with Comparison at T0 and T1**	**t**	**df**	**Sig. (2-tailed)**	**Lower**	**Upper**	**Mean**	**Std. Dev**	**Std. Error Mean**
Pair 1	Sociality Area Scale P0 - P1	6,718	52	,000	1,91	3,53	-2,72	2,94	0,40
Pair 2	Sociality Area Scale C0 - C1	5,916	29	,000	0,96	1,97	-1,47	1,36	0,25
Pair 3	Culture and Knowledge P0 - P1	4,045	52	,000	0,79	2,34	-1,57	2,82	0,39
Pair 4	Culture and Knowledge C0 - C1	4,166	48	,000	0,67	1,91	-1,29	2,16	0,31
Pair 5	Housing and Daily Life Scale P0-P1	6,981	51	,000	3,15	5,70	-4,42	4,57	0,63
Pair 6	Housing and Daily Life Scale C0-C1	2,40	44	,021	0,21	2,37	-1,29	3,60	0,54
Pair 7	Income and Employment Scale P0 - P1	4,752	52	,000	1,14	2,82	-1,98	3,04	,417
Pair 8	Income and Employment Scale C0 - C1	1,917	42	,062	-,020	,076	-,37	1,28	,194

**Table 5:** Residential facilities and living conditions of the project beneficiaries at the T2 December 2019.

-	**Surviving Beneficiaries**		**Deceased Beneficiaries**	
Place	N.	*%*	N.	*%*
Living at home	16	*36,4*	4	*36,4*
Living in a therapeutic community (TC)	17	*38,6*	6	*54,5*
Community housing or group-apartment	8	*18,2*	1	*9,1*
Residences for the Execution of Security Measures (REMS) or prison	3	*6,8*	0	*0*
Total	44	*100*	11	*100*

## Data Availability

Not applicable.

## LIST OF ABBREVIATIONS

FPH: Forensic Psychiatric Hospital
RESM: Residences for the Execution of Security Measures
CA: Capability Approach
PCB: Personal Capability Budget
CFM: Community Foundation of Messina
REMS: Residential structures with high control intensity
TC: Therapeutic Community
CA: Capability Approach
CRPD: Convention on the Rights of Persons with Disabilities
